# Diethyl 2,2′-(ethane-1,2-diyldi­oxy)di­benzo­ate

**DOI:** 10.1107/S1600536814007673

**Published:** 2014-04-12

**Authors:** Huaduan Shi, Haisha Qin, Zhen Ma

**Affiliations:** a School of Chemistry and Chemical Engeneering, Guangxi University, Guangxi 530004, People’s Republic of China

## Abstract

The mol­ecular title compound, C_20_H_22_O_6_, was obtained by the reaction of ethyl 2-hy­droxy­benzoate with 1,2-di­chloro­ethane. The mol­ecule lies on a twofold rotation axis which passes through the middle of the central ethyl­ene bridge. This group exhibits a *gauche* conformation with the corresponding O—C—C—O torsion angle being 73.2 (2)°. The C atoms of the carboxyl group, the aryl and the O—CH_2_ group are coplanar, with an r.m.s. deviation of 0.01 Å. The two aryl rings form a dihedral angle of 67.94 (4)°. The ester ethyl group is disordered over two sets of sites with an occupancy ratio of 0.59 (2):0.41 (2). The crystal packing is dominated by van der Waals forces.

## Related literature   

For synthesis and structures of diesters, see: Ma *et al.* (2012 ▶); Hou & Kan (2007 ▶). For properties and applications of diesters, see: Chen & Liu (2002 ▶). For the synthesis of the title compound, see: Ma & Liu (2002 ▶). For standard bond lengths, see: Allen *et al.* (1987 ▶). For background to the applications of organic acids and esters, see: Chanthapally *et al.* (2012 ▶); Yan *et al.* (2012 ▶).
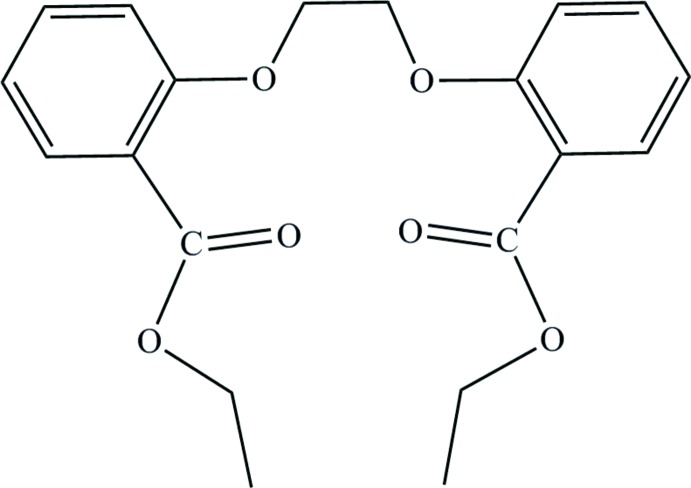


## Experimental   

### 

#### Crystal data  

C_20_H_22_O_6_
*M* *_r_* = 358.38
Orthorhombic, 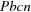

*a* = 21.805 (4) Å
*b* = 9.871 (2) Å
*c* = 8.8646 (18) Å
*V* = 1908.0 (6) Å^3^
*Z* = 4
Mo *K*α radiation
μ = 0.09 mm^−1^
*T* = 298 K
0.35 × 0.31 × 0.28 mm

#### Data collection  

Bruker SMART CCD diffractometer
Absorption correction: multi-scan (*SADABS*; Bruker, 2002 ▶) *T*_min_ = 0.858, *T*_max_ = 1.000
11280 measured reflections
2192 independent reflections
1543 reflections with *I* > 2σ(*I*)
*R*_int_ = 0.023

#### Refinement  

*R*[*F* ^2^ > 2σ(*F* ^2^)] = 0.041
*wR*(*F* ^2^) = 0.132
*S* = 1.04
2192 reflections
140 parameters
24 restraints
H-atom parameters constrained
Δρ_max_ = 0.17 e Å^−3^
Δρ_min_ = −0.14 e Å^−3^

### 

Data collection: *SMART* (Bruker, 2002 ▶); cell refinement: *SAINT* (Bruker, 2002 ▶); data reduction: *SAINT*; program(s) used to solve structure: *SHELXS97* (Sheldrick, 2008 ▶); program(s) used to refine structure: *SHELXL97* (Sheldrick, 2008 ▶); molecular graphics: *SHELXTL* (Sheldrick, 2008 ▶); software used to prepare material for publication: *SHELXL97*.

## Supplementary Material

Crystal structure: contains datablock(s) I, dierster-0. DOI: 10.1107/S1600536814007673/wm5015sup1.cif

Structure factors: contains datablock(s) I. DOI: 10.1107/S1600536814007673/wm5015Isup2.hkl

Supporting information file. DOI: 10.1107/S1600536814007673/wm5015Isup3.cml

CCDC reference: 995747

Additional supporting information:  crystallographic information; 3D view; checkCIF report

## Figures and Tables

**Fig. 1.**
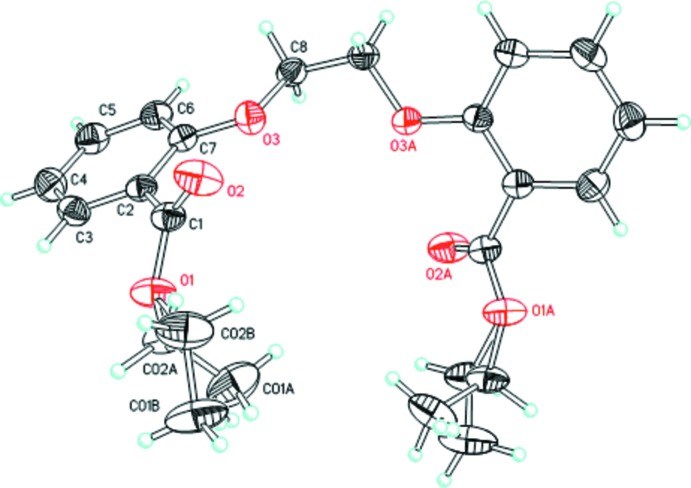
The molecular structure of the title compound with the atom numbering scheme. Displacement ellipsoids are drawn at the 30% probability level. H atoms are presented as small spheres of arbitrary radius. [symmetry code: (A) 1-x, y, 1/2-z ]

**Fig. 2.**
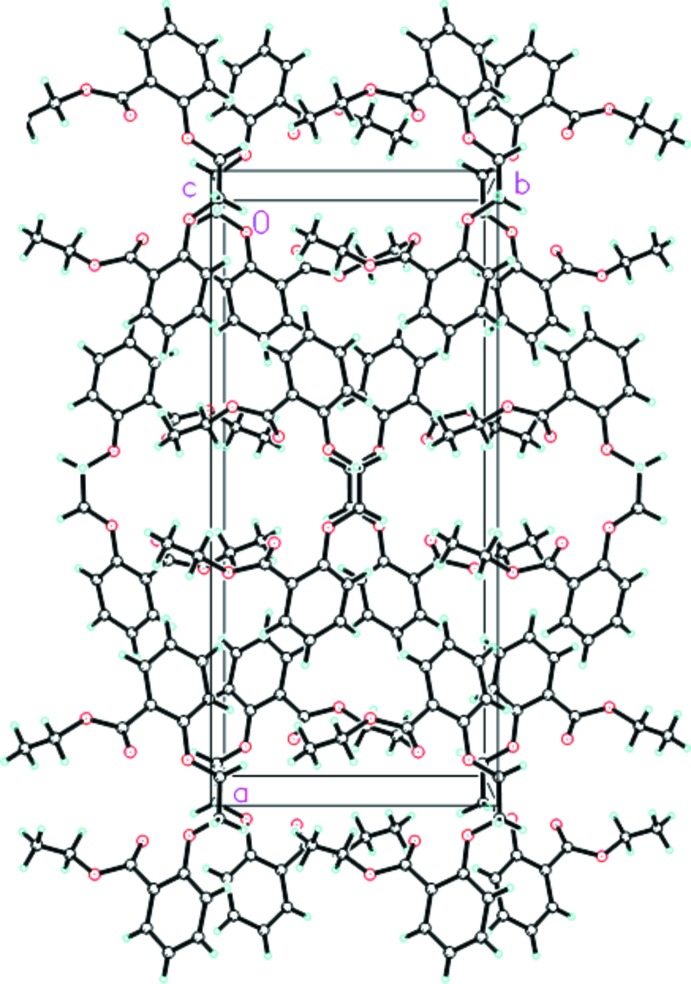
A view of the crystal packing along the c axis.

## supplementary crystallographic information

### 1. Comment

In recent years the chemistry of carboxylic compounds has been the subject of intense studies because of the potential applications of these compounds as ligands for metal complexes or of potential applications as luminescent, non-linear optical, electrical conductive and liquid-crystalline materials (Yan *et al.*, 2012. Chanthapally *et al.*, 2012). Esters are also very important since these compounds are commodity chemicals used as intermediates in the manufacture of acids and in the production of numerous important industrial products. Hence, the current work aims to synthesize new esters for acid production and for investigation of their coordination behaviors with metal ions (Ma *et al.*, 2012; Chen & Liu, 2002). Here, we report the crystal structure of a new diester, C_20_H_22_O_6_, which was obtained by reaction of ethyl 2-hydroxybenzoate with 1,2-dichloroethane.

The structure of C_20_H_22_O_6_ consists of a neutral molecular unit (Fig. 1). The molecule lies on a twofold rotation axis which passes through the middle of the central ethylene bridge that has a *gauche* conformation with the corresponding O—C—C—O torsion angle being 73.2 (2) °. All bond lengths and angles are within normal ranges (Allen *et al.*, 1987). The carbon atom of the carboxyl group, and the aryl and O—CH_2_ moeities of one half molecule are coplanar with an r.m.s. deviation of 0.01 Å. The two aryl rings form a dihedral angle of 67.94 (4) °. The ester ethyl group is disordered over two sets of sites in a 0.59 (2):0.41 (2) occupancy ratio. The packing of the molecules in the crystal structure is shown in Fig. 2.

### 2. Experimental

The title compound was obtained by the reaction of ethyl 2- hydroxybenzoate with 1,2-dichloroethane in *N*,*N*'- dimethylformamide (DMF) according to a reported procedure (Ma & Liu, 2002). In a 100 cm^3^ flask fitted with a funnel, ethyl 2- hydroxybenzoate (8.3 g, 50 m*M*) and potassium carbonate were mixed in 50 cm^3^ of DMF. To this solution was added dropwise a stoichiometric quantity of 1,2-dichloroethane (2.5 g, 25 m*M*) dissolved in 20 cm^3^ of DMF for a period of an hour under stirring. The mixture was further stirred for 24 h at 353 K. The solution was concentrated under reduced pressure and the white solid precipitated by adding a large quantity of water (200 cm^3^) was filtered off and recrystallized from ethanol and decolored with activated carbon. A colorless solid was finally obtained (yield 81 %, m.p: 417–419 K). Slow evaporation of a solution of the title compound in ethanol and dichloromethane (1:1) led to the formation of colorless crystals, which were suitable for X-ray characterization.

### 3. Refinement

All H atoms were positioned geometrically and refined using a riding model with C—H = 0.93 - 0.97 Å and with *U*_iso_(H) = 1.2 times *U*_eq_(C) or 1.5 times *U*_eq_ (methyl C). The two carbon atoms of the ethyl group are disordered over two sets of sites with an occupancy ratio of 0.59 (2):0.41 (2). The C atoms of this group were additionally refined with the ISOR command in SHELXL.

### Crystal data

**Table:**

C_20_H_22_O_6_	*F*(000) = 760
*M**_r_* = 358.38	*D*_x_ = 1.248 Mg m^−^^3^
Orthorhombic, *P**b**c**n*	Mo *K*α radiation, λ = 0.71073 Å
Hall symbol: -P 2n 2ab	Cell parameters from 11280 reflections
*a* = 21.805 (4) Å	θ = 1.9–27.6°
*b* = 9.871 (2) Å	µ = 0.09 mm^−^^1^
*c* = 8.8646 (18) Å	*T* = 298 K
*V* = 1908.0 (6) Å^3^	Prism, colourless
*Z* = 4	0.35 × 0.31 × 0.28 mm

### Data collection

**Table:**

Bruker SMART CCD diffractometer	2192 independent reflections
Radiation source: fine-focus sealed tube	1543 reflections with *I* > 2σ(*I*)
Graphite monochromator	*R*_int_ = 0.023
Detector resolution: 0 pixels mm^-1^	θ_max_ = 27.6°, θ_min_ = 1.9°
phi and ω scans	*h* = −28→27
Absorption correction: multi-scan (*SADABS*; Bruker, 2002)	*k* = −10→12
*T*_min_ = 0.858, *T*_max_ = 1.000	*l* = −10→11
11280 measured reflections	

### Refinement

**Table:**

Refinement on *F*^2^	Secondary atom site location: difference Fourier map
Least-squares matrix: full	Hydrogen site location: inferred from neighbouring sites
*R*[*F*^2^ > 2σ(*F*^2^)] = 0.041	H-atom parameters constrained
*wR*(*F*^2^) = 0.132	*w* = 1/[σ^2^(*F*_o_^2^) + (0.0606*P*)^2^ + 0.335*P*] where *P* = (*F*_o_^2^ + 2*F*_c_^2^)/3
*S* = 1.04	(Δ/σ)_max_ < 0.001
2192 reflections	Δρ_max_ = 0.17 e Å^−^^3^
140 parameters	Δρ_min_ = −0.14 e Å^−^^3^
24 restraints	Extinction correction: *SHELXL97* (Sheldrick, 2008), Fc^*^=kFc[1+0.001xFc^2^λ^3^/sin(2θ)]^-1/4^
Primary atom site location: structure-invariant direct methods	Extinction coefficient: 0.0102 (19)

### Special details

**Table:**

Geometry. All esds (except the esd in the dihedral angle between two l.s. planes) are estimated using the full covariance matrix. The cell esds are taken into account individually in the estimation of esds in distances, angles and torsion angles; correlations between esds in cell parameters are only used when they are defined by crystal symmetry. An approximate (isotropic) treatment of cell esds is used for estimating esds involving l.s. planes.
Refinement. Refinement of F^2^ against ALL reflections. The weighted R-factor wR and goodness of fit S are based on F^2^, conventional R-factors R are based on F, with F set to zero for negative F^2^. The threshold expression of F^2^ > 2sigma(F^2^) is used only for calculating R-factors(gt) etc. and is not relevant to the choice of reflections for refinement. R-factors based on F^2^ are statistically about twice as large as those based on F, and R- factors based on ALL data will be even larger.

### Fractional atomic coordinates and isotropic or equivalent isotropic displacement parameters (Å^2^)

**Table:**

	*x*	*y*	*z*	*U*_iso_*/*U*_eq_	Occ. (<1)
O1	0.36609 (7)	0.94335 (11)	0.09160 (14)	0.0784 (4)	
O2	0.41410 (7)	0.77861 (13)	−0.03024 (15)	0.0880 (5)	
O3	0.43658 (5)	0.59258 (11)	0.20505 (13)	0.0632 (3)	
C1	0.38057 (7)	0.81417 (15)	0.06775 (17)	0.0570 (4)	
C02A	0.3859 (9)	1.0477 (15)	−0.0051 (11)	0.075 (3)	0.41 (2)
H02A	0.4024	1.0099	−0.0976	0.090*	0.41 (2)
H02B	0.3518	1.1066	−0.0307	0.090*	0.41 (2)
C02B	0.4008 (7)	1.0397 (13)	−0.0105 (13)	0.114 (4)	0.59 (2)
H02C	0.4421	1.0064	−0.0273	0.137*	0.59 (2)
H02D	0.3804	1.0466	−0.1074	0.137*	0.59 (2)
C2	0.34747 (7)	0.72278 (14)	0.17329 (15)	0.0522 (4)	
C01A	0.4309 (8)	1.120 (2)	0.0708 (15)	0.127 (4)	0.41 (2)
H01A	0.4127	1.1683	0.1536	0.191*	0.41 (2)
H01B	0.4497	1.1835	0.0029	0.191*	0.41 (2)
H01C	0.4614	1.0587	0.1084	0.191*	0.41 (2)
C01B	0.4030 (7)	1.1719 (7)	0.0618 (8)	0.119 (3)	0.59 (2)
H01D	0.3620	1.2059	0.0738	0.179*	0.59 (2)
H01E	0.4262	1.2333	0.0004	0.179*	0.59 (2)
H01F	0.4220	1.1637	0.1590	0.179*	0.59 (2)
C3	0.28631 (8)	0.74645 (17)	0.20651 (18)	0.0654 (4)	
H3A	0.2668	0.8215	0.1650	0.078*	
C4	0.25382 (8)	0.6610 (2)	0.3000 (2)	0.0766 (5)	
H4A	0.2126	0.6773	0.3198	0.092*	
C5	0.28283 (8)	0.55161 (18)	0.3634 (2)	0.0730 (5)	
H5A	0.2611	0.4946	0.4275	0.088*	
C6	0.34359 (7)	0.52495 (15)	0.33381 (19)	0.0634 (4)	
H6A	0.3627	0.4505	0.3778	0.076*	
C7	0.37642 (7)	0.60994 (14)	0.23769 (16)	0.0518 (4)	
C8	0.46777 (7)	0.48215 (15)	0.27597 (19)	0.0609 (4)	
H8A	0.4482	0.3972	0.2493	0.073*	
H8B	0.4662	0.4922	0.3848	0.073*	

### Atomic displacement parameters (Å^2^)

**Table:**

	*U*^11^	*U*^22^	*U*^33^	*U*^12^	*U*^13^	*U*^23^
O1	0.1251 (11)	0.0471 (6)	0.0631 (7)	0.0001 (6)	0.0089 (7)	0.0031 (5)
O2	0.1222 (11)	0.0685 (8)	0.0732 (8)	0.0098 (7)	0.0363 (8)	0.0084 (6)
O3	0.0597 (6)	0.0607 (6)	0.0692 (7)	0.0009 (5)	0.0008 (5)	0.0183 (5)
C1	0.0753 (9)	0.0505 (8)	0.0451 (7)	0.0019 (7)	−0.0047 (7)	−0.0018 (6)
C02A	0.128 (7)	0.054 (4)	0.043 (3)	−0.014 (4)	−0.014 (4)	0.008 (3)
C02B	0.174 (9)	0.065 (4)	0.104 (6)	−0.006 (4)	0.034 (5)	0.020 (4)
C2	0.0654 (9)	0.0485 (7)	0.0425 (7)	−0.0015 (6)	−0.0024 (6)	−0.0060 (6)
C01A	0.146 (10)	0.103 (9)	0.133 (7)	−0.059 (7)	0.022 (6)	0.010 (7)
C01B	0.228 (10)	0.057 (3)	0.073 (3)	−0.031 (4)	−0.001 (4)	0.003 (2)
C3	0.0717 (10)	0.0647 (9)	0.0597 (9)	0.0118 (8)	0.0008 (8)	−0.0057 (8)
C4	0.0655 (10)	0.0869 (12)	0.0774 (12)	−0.0004 (9)	0.0129 (9)	−0.0070 (10)
C5	0.0767 (11)	0.0687 (10)	0.0735 (11)	−0.0147 (9)	0.0173 (9)	0.0001 (9)
C6	0.0733 (10)	0.0530 (8)	0.0640 (9)	−0.0065 (7)	0.0039 (8)	0.0054 (7)
C7	0.0580 (8)	0.0487 (7)	0.0488 (8)	−0.0043 (6)	−0.0011 (6)	−0.0018 (6)
C8	0.0677 (8)	0.0487 (8)	0.0662 (9)	−0.0034 (7)	−0.0087 (7)	0.0060 (7)

### Geometric parameters (Å, º)

**Table:**

O1—C1	1.3306 (18)	C01A—H01B	0.9600
O1—C02A	1.408 (14)	C01A—H01C	0.9600
O1—C02B	1.516 (14)	C01B—H01D	0.9600
O2—C1	1.1884 (19)	C01B—H01E	0.9600
O3—C7	1.3541 (18)	C01B—H01F	0.9600
O3—C8	1.4304 (17)	C3—C4	1.379 (2)
C1—C2	1.487 (2)	C3—H3A	0.9300
C02A—C01A	1.39 (2)	C4—C5	1.372 (3)
C02A—H02A	0.9700	C4—H4A	0.9300
C02A—H02B	0.9700	C5—C6	1.376 (2)
C02B—C01B	1.455 (15)	C5—H5A	0.9300
C02B—H02C	0.9700	C6—C7	1.394 (2)
C02B—H02D	0.9700	C6—H6A	0.9300
C2—C3	1.386 (2)	C8—C8^i^	1.479 (3)
C2—C7	1.402 (2)	C8—H8A	0.9700
C01A—H01A	0.9600	C8—H8B	0.9700
			
C1—O1—C02A	122.1 (7)	C02B—C01B—H01F	109.5
C1—O1—C02B	112.8 (5)	H01D—C01B—H01F	109.5
C02A—O1—C02B	12.6 (12)	H01E—C01B—H01F	109.5
C7—O3—C8	117.59 (11)	C4—C3—C2	121.27 (16)
O2—C1—O1	123.04 (15)	C4—C3—H3A	119.4
O2—C1—C2	125.41 (14)	C2—C3—H3A	119.4
O1—C1—C2	111.49 (13)	C5—C4—C3	119.41 (16)
C01A—C02A—O1	107.4 (10)	C5—C4—H4A	120.3
C01A—C02A—H02A	110.2	C3—C4—H4A	120.3
O1—C02A—H02A	110.2	C4—C5—C6	121.09 (16)
C01A—C02A—H02B	110.2	C4—C5—H5A	119.5
O1—C02A—H02B	110.2	C6—C5—H5A	119.5
H02A—C02A—H02B	108.5	C5—C6—C7	119.74 (15)
C01B—C02B—O1	108.4 (10)	C5—C6—H6A	120.1
C01B—C02B—H02C	110.0	C7—C6—H6A	120.1
O1—C02B—H02C	110.0	O3—C7—C6	123.51 (13)
C01B—C02B—H02D	110.0	O3—C7—C2	116.73 (12)
O1—C02B—H02D	110.0	C6—C7—C2	119.73 (14)
H02C—C02B—H02D	108.4	O3—C8—C8^i^	108.37 (12)
C3—C2—C7	118.74 (14)	O3—C8—H8A	110.0
C3—C2—C1	119.91 (13)	C8^i^—C8—H8A	110.0
C7—C2—C1	121.32 (13)	O3—C8—H8B	110.0
C02B—C01B—H01D	109.5	C8^i^—C8—H8B	110.0
C02B—C01B—H01E	109.5	H8A—C8—H8B	108.4
H01D—C01B—H01E	109.5		
			
C02A—O1—C1—O2	4.9 (7)	C1—C2—C3—C4	177.81 (15)
C02B—O1—C1—O2	−4.7 (6)	C2—C3—C4—C5	1.1 (3)
C02A—O1—C1—C2	−172.4 (7)	C3—C4—C5—C6	−0.8 (3)
C02B—O1—C1—C2	177.9 (6)	C4—C5—C6—C7	−0.1 (3)
C1—O1—C02A—C01A	−107.6 (14)	C8—O3—C7—C6	−1.2 (2)
C02B—O1—C02A—C01A	−63 (4)	C8—O3—C7—C2	176.78 (13)
C1—O1—C02B—C01B	−156.1 (10)	C5—C6—C7—O3	178.61 (15)
C02A—O1—C02B—C01B	64 (4)	C5—C6—C7—C2	0.7 (2)
O2—C1—C2—C3	−136.02 (18)	C3—C2—C7—O3	−178.45 (13)
O1—C1—C2—C3	41.24 (19)	C1—C2—C7—O3	3.3 (2)
O2—C1—C2—C7	42.3 (2)	C3—C2—C7—C6	−0.4 (2)
O1—C1—C2—C7	−140.48 (14)	C1—C2—C7—C6	−178.72 (13)
C7—C2—C3—C4	−0.5 (2)	C7—O3—C8—C8^i^	179.76 (14)

Symmetry code: (i) −*x*+1, *y*, −*z*+1/2.
